# A Systematic Microfluidic Study of the Use of Diluted Silica Sols to Enhance Oil Displacement

**DOI:** 10.3390/nano14141233

**Published:** 2024-07-22

**Authors:** Andrey I. Pryazhnikov, Maxim I. Pryazhnikov, Alexander S. Lobasov, Andrey V. Minakov

**Affiliations:** 1 Laboratory of Physical and Chemical Technologies for the Development of Hard-to-Recover Hydrocarbon Reserves, Siberian Federal University, 660041 Krasnoyarsk, Russia; apryazhnikov@sfu-kras.ru (A.I.P.);; 2 Laboratory of Heat Exchange Control in Phase and Chemical Transformations, Kutateladze Institute of Thermophysics, 630090 Novosibirsk, Russia

**Keywords:** enhanced oil recovery, microfluidics, nanoparticle suspension, crude oil, microfluidic chip, displacement fluid

## Abstract

The paper presents the results of a systematic microfluidic study of the application of nanosuspensions for enhanced oil recovery. For the first time, approximately a dozen nanosuspensions prepared by the dilution of silica sols as displacement fluids were considered. The concentration of nanoparticles in the suspensions varied from 0.125 to 2 wt%, and their size ranged from 10 to 35 nm. Furthermore, the silica sols under consideration differed in their compositions of functional groups and pH. The effects of concentration, nanoparticle size, fluid flow rate, and the viscosity of the displaced oil were investigated using microfluidic technology. The microfluidic experiments demonstrated that the application of nanosuspensions for water flooding has significant potential. The efficiency of oil displacement by nanosuspensions was found to increase significantly (up to 30%) with the increasing concentration and decreasing average size of nanoparticles. The application of nanosuspensions for the enhancement of oil recovery is most appropriate for reservoirs with highly viscous oil.

## 1. Introduction

One of the promising avenues of research in the field of oil recovery is the utilization of microfluidic chips for the modeling of flows within porous media. Microchannel chips offer a number of advantages over traditional research methods. These advantages include a low consumption of components, the possibility of visual observation of oil displacement processes, the possibility of reusing microchips, a short experiment time, a compact size and low energy consumption of the experimental facility, the possibility of flexible parameter tuning, the absence of interaction between components and the environment, and others. It is not surprising that numerous studies of enhanced oil recovery employ microfluidic chips.

Thus, the authors of study [1,2,3] evaluated the advantages of such chips over conventional methodologies and investigated the impact of injection rate, microfluidic model structure, oil type, and other parameters on efficiency of oil recovery. Consequently, it was demonstrated that emulsification and mixing increased with an elevated injection rate. Furthermore, it was demonstrated that brine with a low salinity permitted the recovery of a greater quantity of oil than brine with a high salinity. As a result, the authors were able to develop a reliable and reproducible microfluidic flooding procedure.

Yun et al. [4] explored the possibility of utilizing microfluidic chips to assess oil recovery efficacy, with a focus on the effect of interfacial tension and surface wettability. It has been demonstrated that the utilization of microfluidic chips can result in a notable reduction in research costs and the time required for experimentation.

In work [5], the impact of wettability on oil recovery was investigated. Glass micromodels were employed to simulate quartz surfaces of hydrophilic porous rocks (e.g., sandstone), and hydrophobic coatings were utilized to alter their wettability. It has been demonstrated that alterations in wettability have a pronounced impact on oil recovery efficiency. Moreover, it has been demonstrated that flooding with surfactants is a more effective method than using brine.

The authors of [6] conducted an experimental study to assess the potential of microfluidic technologies to enhance oil displacement efficiency. The advantages of the polymethyl methacrylate chip, developed by the authors of this work, include its rapid fabrication and low cost. The effect of the flow rate of the displacement agent, which was water, on the oil recovery factor was examined. It has been demonstrated that increasing the flow rate by 20 times (from 25 to 500 μL/min) leads to an increase in the oil recovery factor by 23%. In this case, the dependence of the oil recovery factor on flow rate is nonlinear.

Despite the fact that to date, the primary displacement agent remains water of varying salinity, the number of studies considering the potential use of other liquids or gases is on the rise. For instance, research [7] illustrates the potential of utilizing carbon dioxide as a displacement agent. Concurrently, the authors posit that enhanced oil recovery can simultaneously mitigate carbon dioxide emissions into the atmosphere. This is achieved by sequestering the gas in oil-bearing formations.

The results of the conducted studies indicate that the use of nanofluids can enhance oil recovery in the majority of cases. In a number of studies, various surfactants and polymers [8,9] as well as nanofluids [10,11,12] have been employed for the displacement of oil from microfluidic chips. In all cases, it has been demonstrated that their use enhances the efficiency of oil recovery compared to water flooding.

It is the use of nanoparticles (in the broad sense) that is currently a prominent focus of numerous studies investigating the potential for enhancing oil recovery efficiency through the use of microfluidic devices. For example, the authors of [13] conducted an experimental investigation into the efficacy of silica-based nanofluid in enhancing oil recovery efficiency through a lab-on-a-chip approach. It has been demonstrated that a nanofluid comprising nanoparticles at a concentration of 2 wt% and an average size of approximately 22 nm can enhance the oil recovery factor by 16% in comparison to conventional water.

Rostami et al. [14] examined the effect of silica nanoparticles on the alteration in wettability of glass micromodels both numerically and experimentally. Experiments were conducted on both water-wetted and oil-wetted surfaces. The results were then compared with those obtained through the pumping of water through the microchip. As a result, it was determined that the initial wettability of the system plays a pivotal role in determining the ultimate oil recovery. Furthermore, it was determined that the alteration in wettability resulting from the water flooding of the microchip with nanofluid is a time-dependent process. This phenomenon can be attributed to the adsorption of silica nanoparticles on the solid surface, which subsequently led to the formation of a nano-texture coating. This phenomenon alters the surface wettability from oil-wettable to intermediate-wettable. The ORF in this case enhanced by approximately 10%. Furthermore, the simulation results have been shown to be in good agreement with experimental data.

The authors of the work [15] conducted a study to investigate the effect of the weight concentration of silica nanoparticles with an average size of 22 nm on the oil recovery factor when pumping a displacement agent through a microchip made of glass. Such microchip design allowed visual observations of the oil displacement process. As a result, nonlinear dependencies of breakthrough time and oil recovery factor on nanoparticle weight concentration were identified. It was demonstrated that an increase in the concentration from 0 to 2 wt% resulted in a 27% enhancement in the oil recovery factor (from 60 to 76%). Moreover, a pronounced effect of nanofluid utilization was observed even at a nanoparticle concentration of 0.5 wt%.

Despite the current prevalence of microfluidics in the chemical methods applied for oil recovery enhancement, there is a paucity of systematic data on the use of nanosuspensions for these purposes. This paper presents the results of a first-time systematic microfluidics study on the performance efficiency of about a dozen nanosuspensions prepared by diluting silica solutions to be used as displacement fluids. The effect of nanosuspension concentration, particle size, pH, and oil viscosity on the oil recovery factor was investigated. This is the main novelty of this work. The results of such a comprehensive microfluidic study of nanosuspensions were previously unavailable. The results of microfluidic experiments have demonstrated that nanosuspensions have significant potential for application in water flooding. Their efficacy is contingent upon concentration and size of nanoparticles, as well as the composition of functional groups. It is supposed that they will be most effective in formations with more viscous oil.

## 2. Materials and Methods

### 2.1. Crude Oil

Three oil samples, designated as O1, O2, and O3 and differing in density and viscosity, were used in this study. The two light crude oils (API gravity of 38.5° and 34.0°) and one medium crude oil (25.0°) were used according to ASTM D287-22 [16]. The viscosity of the oil samples was measured by means of a rotary viscometer at a temperature of 25 °C. The density of the oil was determined by the pycnometer method. The error in determining density by this method did not exceed 0.1 kg/m^3^.

The properties of the various crude oil samples are presented in Table 1. Samples of crude oil with significantly different viscosities were investigated.

The elemental analysis of oil samples by X-ray fluorescence spectrometer Axios^mAX^-Petro (PANalytical, Almelo, The Netherlands) [17,18] is presented in Table 2. X-ray fluorescence analysis uses a method based on the principle of measuring the spectrum of secondary X-ray radiation. The primary X-rays produced by the X-ray tube irradiate the sample being analyzed and produce secondary X-rays, the spectrum of which depends on the elemental composition of the sample. An X-ray tube is used as an excitation source. Calculation of the mass fraction of the analyzed elements is based on the dependence of the radiation intensity on its mass fraction in the sample. The O1 and O3 samples exhibited high chlorine content. All samples exhibited a considerable quantity of sulfur. Additionally, the presence of calcium and barium suggests high inorganic chlorine content.

**Figure 1:**
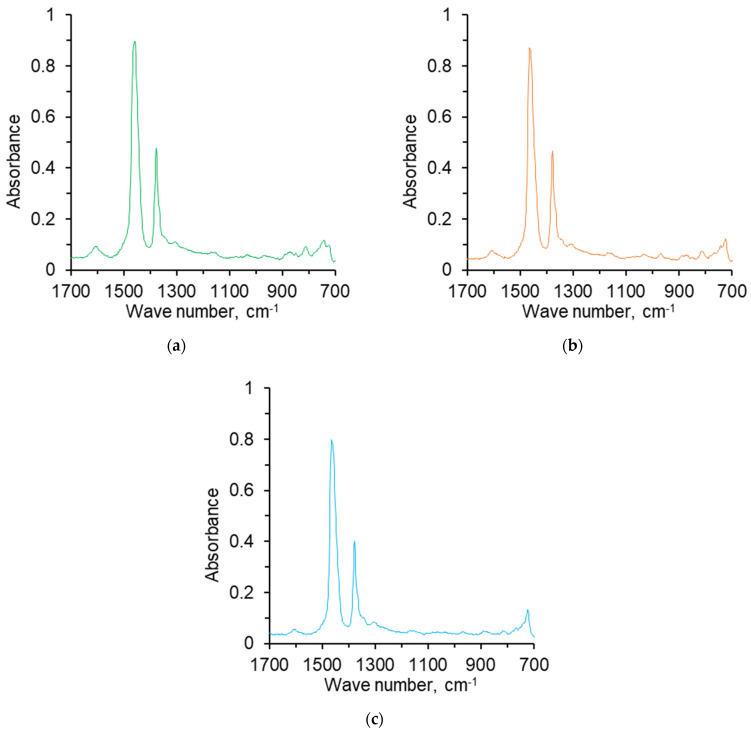
IR spectrum of the oil. (**a**) Sample O1; (**b**) Sample O2; (**c**) Sample O3.

The composition of various organic and inorganic substances and their compounds was determined through Fourier transform infrared spectroscopy (FTIR). The FTIR method is based on the microscopic interaction of infrared light with a chemical substance through an absorption process, which results in the production of a spectrum unique to the chemical substance, serving as a “molecular fingerprint”. Figure 1 depicts the absorption spectra of a variety of oil samples obtained via FTIR. The optical density at the maximum of the absorption bands, as well as the following spectral coefficients, were determined (see Table 3): C_1_ = D_1600_/D_720_ (aromaticity); C_2_ = D_1710_/D_1465_ (oxidation); C_3_ = D_1380_/D_1465_ (branching); C_4_ = (D_720_ + D_1380_)/D_1600_ (aliphaticity), and C_5_ = D_1030_/D_1465_ (sulfation).

The oil samples can be described as follows: sample O3 is a methane oil, sample O2 is a methane-naphthenic oil, and sample O1 is a naphthenic oil.

### 2.2. Nanoparticle Suspensions

In this study, water-based nanosuspensions were prepared by diluting concentrated silica sols in distilled water. Highly concentrated sols were obtained by dissolving sodium silicate in water to obtain sodium liquid glass. This was followed by ion exchange processes accompanied by simultaneous ash formation and thermal stabilization.

Table 4 presents a brief description of the initial samples in accordance with specifications provided by their manufacturer (LLC Russilica, Moscow, Russia), which were utilized in the preparation of nanosuspensions for subsequent analysis. The initial sols were aqueous dispersions of silica, which differed in composition, stabilization method, and size of the dispersed phase. In 1030 and 3550 silica sols, the nanoparticles exhibited a negative surface charge, which was stabilized by alkali metal ions (Na^+^). In 2040AS, ash SiO_2_ nanodispersed powder exhibited a negative surface charge stabilized by the presence of ammonium ions (NH_4_^+^). The silica nanoparticles in WA1530 ash were treated with an aluminum stabilizing component. In addition to the charge of the nanoparticles and their stabilization method, all the sols differed in their hydrogen index. The average particle size of the silica sols ranged from 10 to 35 nm, while their concentration ranged from 30 to 50 wt%. The nanosuspensions under investigation were prepared by diluting the concentrated silica sols with distilled water. The concentrations under investigation spanned a range from 0.125 to 2 wt%.

**Figure 2:**
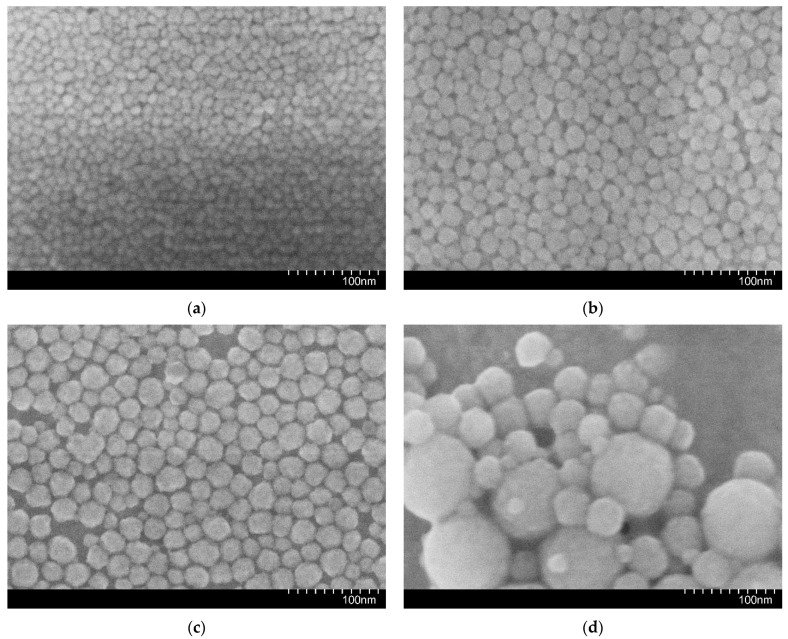
Electron microscopy photographs of (**a**) 1030; (**b**) WA1530; (**c**) 2040AS; and (**d**) 3550 nanoparticles.

Electron microscopic studies were conducted using a high-resolution FE-SEM Hitachi S-5500 scanning electron microscope (Hitachi High-Tech Corporation, Tokyo, Japan). All electron images were captured in secondary electron (SE) mode at an accelerating voltage of 3 kV, a beam current of 10 μA, and a focal length ranging from 100 to 200 μm. Figure 2 illustrates representative electron micrographs of 1030, WA1530, 2040AS, and 3550 nanoparticles.

The results of the scanning electron microscopy (SEM) analysis of the micrographs corroborate the data provided by the manufacturer regarding the average particle size (Table 4). Furthermore, the electron micrographs indicated that the nanoparticles in the 1030, WA1530, and 2040AS nanosuspensions exhibited a narrow nanoparticle size distribution, while the nanosuspension 3550 exhibited a broad particle size distribution.

A crucial property of nanosuspension is its viscosity. Viscosity plays a pivotal role in the challenges associated with the oil recovery enhancement in water flooding of oil fields. The greater the viscosity coefficient, the more effective the use of nanosuspension tends to be. Consequently, it is of great significance to have data on the viscosity coefficient of nanosuspension. The viscosity was quantified using a Brookfield DV2T rotational viscometer (Middleboro, Massachusetts, USA), which employs a calibrated spring to measure the twisting of the spindle as it rotates at a constant speed within the liquid under study. The Brookfield DV2T viscometer is reported to have an error of ±2%, depending on the range used. Given that nanosuspensions frequently exhibit non-Newtonian properties [19], the present study sought to ascertain the dependence of the viscosity coefficient on the shear rate over the entire available range of variation in all cases considered. The temperature was meticulously maintained throughout the measurement process. All data presented below were obtained at 25 °C. As a result, it was determined that within the concentration range in question, all the nanosuspensions under consideration exhibited Newtonian fluid behavior. The viscosity coefficient and density data for the nanosuspensions are presented in Table 5. As is seen, the viscosity coefficient increased with increasing nanoparticle concentration. The maximum increment was approximately 12% in comparison to water. Suspensions prepared from sol 1030 had a higher viscosity than those from other sols at the same concentration. Furthermore, the analysis has demonstrated that the viscosity of nanosuspension increased with decreasing the size of nanoparticles. The minimum viscosity was exhibited by 3550 nanosuspension. This behavior is in good agreement with the current understanding of nanofluids. The relationship between the viscosity coefficient of nanosuspensions and particle size has been extensively studied in [20]. As can be observed, the densities of the nanosuspensions differ slightly from the density of water under the same conditions.

### 2.3. Microfluidic Chip and Experimental Technique

**Figure 3:**
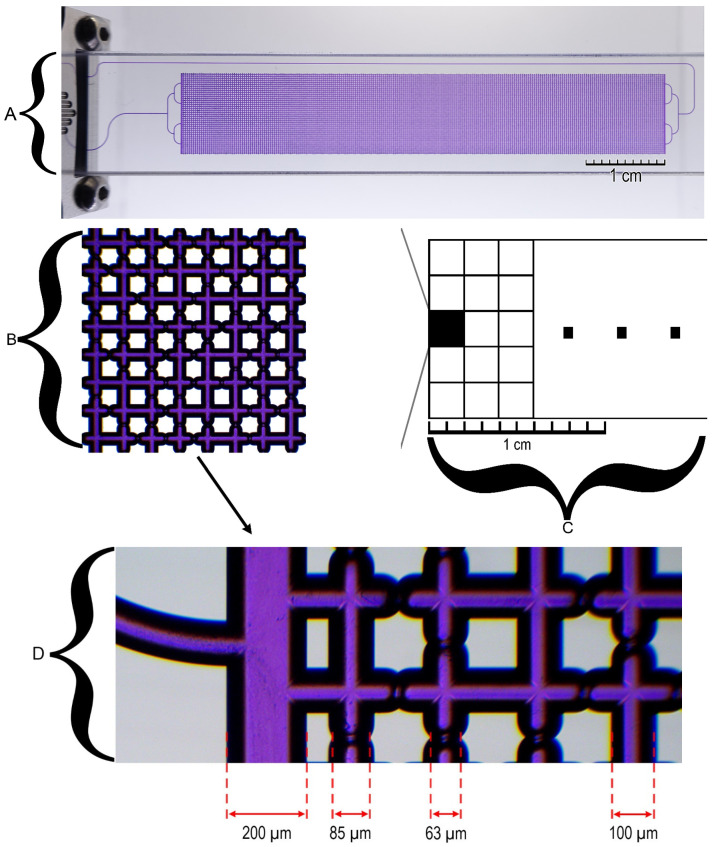
Schematic diagram illustrating the chip structure: (**A**) A porous region in the middle of the chip; (**B**) A square structure measuring 8 × 8 channels; (**C**) A porous structure formed by repeating squares measuring 2 × 2 mm^2^; (**D**) Various types of channels forming a square structure.

In this study, a microfluidic chip was employed to simulate complex porous rock structures with a view to investigating issues pertaining to oil and gas production, environmental testing, and groundwater analysis (Figure 3A). The microfluidic chip was composed of two layers, upper and lower, each with a thickness of 2 mm. The fabrication of the microfluidic chip involved the etching of sodium–lime glass with hydrogen fluoride, followed by heat sealing. The microfluidic chip had a single inlet and outlet. The standard dimensions of the microfluidic chip, excluding connectors, were 92.5 × 15.0 mm^2^, with a thickness of 4 mm. The porous region of the chip was 10 × 60 mm^2^. The length of the inlet channel, inclusive of forking, was 27.7 mm, and the volume of the inlet channel was 0.9 μL. The outlet channel had a length of 99.2 mm and an internal volume of 3.2 μL. The total length of the porous region was 4800 mm, and the volume of the porous region was approximately 38 μL. The surface roughness of the channels was 5 nm. At a water flow rate of 100 μL/min, the back pressure was 1 bar. The porous structure was formed by repeating a 2 × 2 mm^2^ squares 150 times (Figure 3C). The channel grid was a square of 8 × 8 channels (Figure 3B). The constrictions, or “pores”, formed in the channel grid were randomly distributed to simulate the natural structure of the rock. The grid contained three distinct pore types: 38 pores with a diameter of 63 μm, 40 pores with a diameter of 85 μm, and 50 straight channels that had a nearly elliptical cross-section. The channel depth and width were 100 and 110 μm, respectively (Figure 3D).

**Figure 4:**
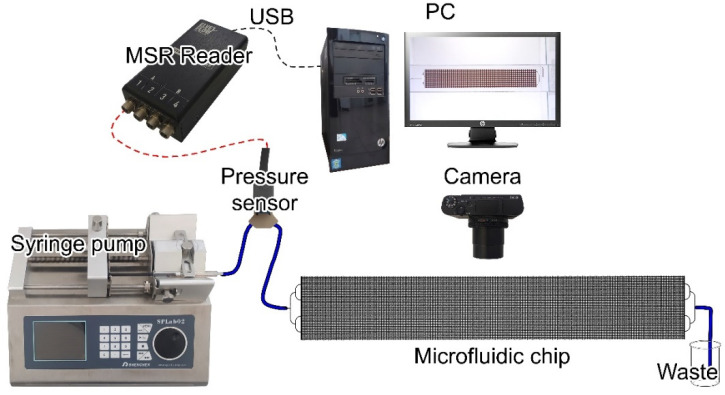
Schematic diagram of the experimental setup.

A detailed description of the experiment is given in [15]. The schematic diagram of the experimental setup is shown in Figure 4. A two-channel syringe pump SPLab02 (Chemyx Inc., Stafford, TX, USA) was used in the experiments (flow rate ranged from 0.831 to 127 mL/min, an error of ± 0.5%, maximum linear motion force 8.5 kg, distance per microstep 0.078 μm, linear velocity ranged from 5 to 132 mm/min). The displacement fluid was supplied via a 1-mL Hamilton syringe with a Luer-type connection. The flow rate was set in milliliters per minute (mL/min), and the pressure versus time was recorded. The pressure drop was monitored and recorded using the Elveflow ESI Microfluidic Software (Elveflow^®^ Smart Interface 3.07.00).

The pressure was recorded using an Elveflow relative pressure sensor (Paris, France) for liquids and gases with an operational range spanning from −15 to 30 psi (2000 mbar). The pressure sensor exhibited the following performance characteristics: a measuring range of 2000 mbar, a minimum and maximum pressure of −15 and 30 psi, respectively, a maximum manometer pressure of 60 psi, a measurement error of no more than ±0.2% of maximum pressure, and a repeatability of ±0.2%. The connector was a pressure screw with replaceable ferrules, thread 10–32. The tube diameter was 1/16″ OD. The wetted materials were polyaryletheretherketone, silicone, and fluorosilicone seal. The operating temperature ranged from −40 to +85 °C. The internal volume was 7.5 µL.

The MSR sensor reader is an interface that was used to collect data from analog sensors, including Elveflow pressure and flow rate sensors. The sensor reader can be utilized to monitor flow, pressure, or other physical parameters employing any type of flow monitoring instrument, including syringe pumps, peristaltic pumps, perfusion pumps, and pressure controllers.

To observe the experiment, a small box was constructed with the interior lined with white matte paper along the walls. Illumination was provided by LED tape. The microfluidic chip was positioned horizontally on the glass. Video and photographic images were captured using a Sony RX100 IV camera (Tokyo, Japan).

### 2.4. Experimental Technique

The empty chip was initially filled with oil, followed by flooding with displacement fluid at a fixed flow rate. Several pore volumes were pumped through the system. The flooding process was recorded by a high-speed camera, which subsequently saved the video for further analysis. Following each experiment, the microfluidic chip was subjected to a series of thorough washes with dichloroethane, isopropanol, distilled water, and purging with air.

The oil recovery factor was evaluated by analyzing photographs obtained during the experiment. The final photographs were obtained by converting the video recording to a format suitable for analysis using the free FFmpeg library (ffmpeg version 5.1.2). The BlackBox Component Builder (Oberon Microsystems, Zurich, Switzerland) application and FreeImage library permit the estimation of the oil recovery factor based on the hue, saturation, and value (HSV) color model. The application enables opening and selecting a region of the microfluidic chip for the purpose of estimating oil pixels. This was achieved by utilizing a threshold based on the specified HSV model. Once the threshold saturation had been determined, normalization was performed with respect to the entire selected area, including background pixels and oil pixels. Batch image processing allowed for the plotting of the time-dependent oil recovery factor. A detailed description of the software operation is presented in [15].

**Figure 5:**
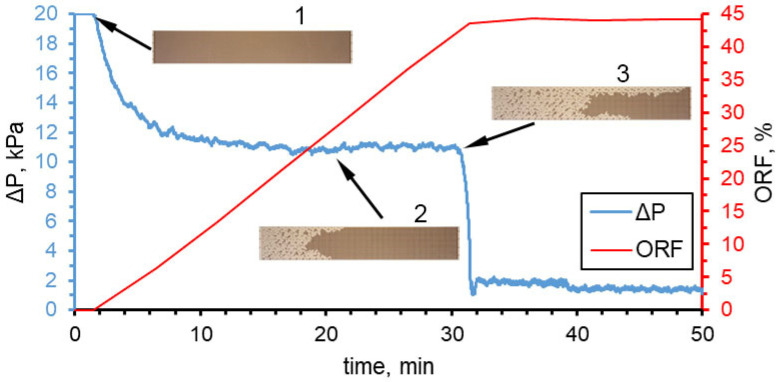
Pressure drop (blue curve) and oil recovery factor (red curve) in the microfluidic chip in the course of displacement: 1—initiation of displacement process, 2—displacement process after 15 min, 3—displacement fluid breakthrough.

In conjunction with the video recording, the pressure drop was recorded in the microfluidic chip. The pressure drop provides supplementary data regarding the filtration of oil from the microfluidic chip. The dependence of pressure drop and oil recovery factor on time is presented in Figure 5. The initial pressure drop in the microfluidic chip was stabilized at a maximum value of approximately 20 kPa. This corresponds to position (1) in Figure 5. Subsequently, the displacement fluid penetrated the porous structure of the microfluidic chip, resulting in a gradual decrease in pressure (position (2) in Figure 5). As the displacement fluid front advanced, the pressure drop stabilized. The point at which the water broke through and reached the outlet of the microfluidic chip is represented by position (3) in Figure 5. At this moment, a precipitous decline in the pressure drop across the microfluidic chip was discernible.

## 3. Research Results and Discussion

### 3.1. Effect of Water Flow Rate (Capillary Number)

**Figure 6:**
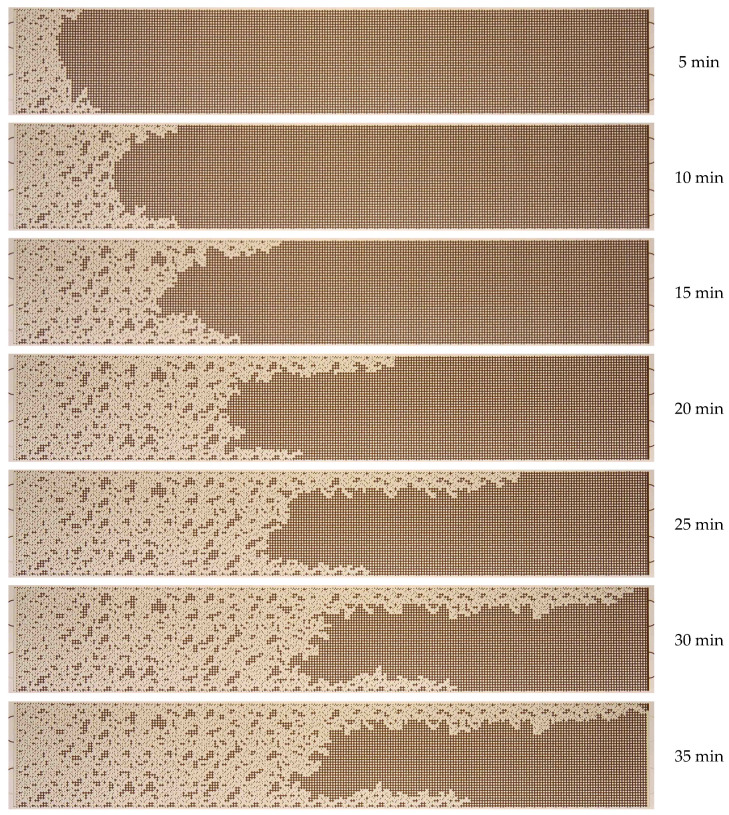
Photographs of the O2 oil displacement process by water over time.

Initially, an experimental microfluidic water flooding procedure was conducted. A series of experiments were performed to investigate the effect of water flow rate on the enhancement of the oil recovery factor. The O2 oil sample was used. A microfluidic chip that was completely filled with oil was fed with displacement water at a given volume flow rate, which ranged from 0.5 to 10 µL/min. This corresponded to a capillary number variation range from 8.4 × 10^−6^ to 1.6 × 10^−4^. The capillary number was defined as the ratio between viscosity and surface tension forces Ca = µ × U/σ. As an illustration, Figure 6 depicts the dynamics of the oil displacement process with water at a flow rate of 0.5 µL/min.

**Figure 7:**
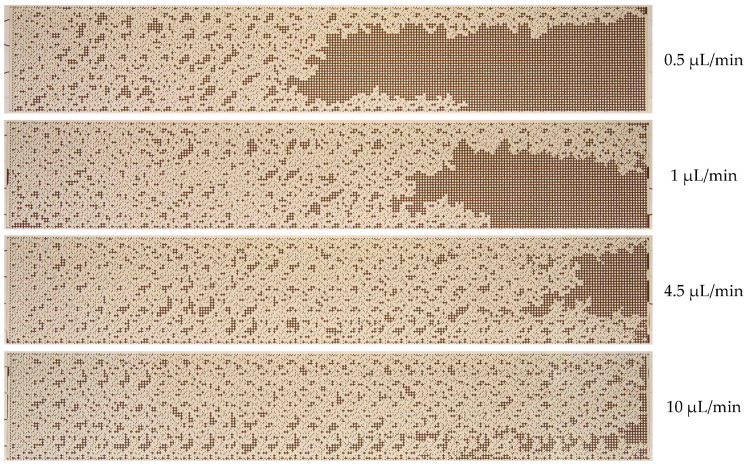
Final photographs of the distribution of O2 oil sample and water after water flooding at different flow rates.

As illustrated in Figure 6, initially, the waterfront exhibits uniform motion, but subsequently, it begins to undergo a transformation. This transformation results from the formation of viscous fingers, which are caused by the unstable water–oil interface. The formation of viscous fingers is a consequence of the lower viscosity of water compared to oil. According to Figure 6, water breaks through to the outlet of the micromodel by approximately 30 min. Up to this point, the oil recovery factor has exhibited a nearly linear increase in proportion to the water flow rate. Following the breakthrough of water, the flow stabilizes, and the oil recovery factor remains practically unchanged. The oil recovery factor in the microfluidic chip, when displacing oil by water, was found to be 44%. Once the oil displacement process has been established (as illustrated in the final photographs in Figure 6), a considerable area remains occupied by oil. Figure 7 depicts the residual oil distribution in the microfluidic chip following the water displacement process at varying flow rates.

**Figure 8:**
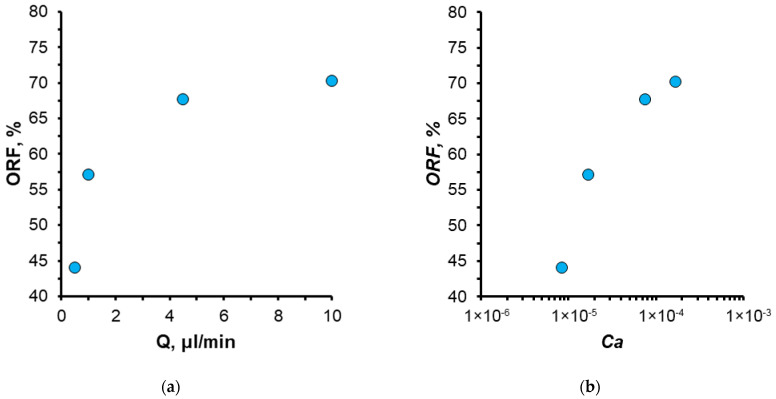
Dependence of oil recovery factor (ORF) of O2 sample on water flow rate (**a**) and capillary number (**b**).

The dependence of the oil recovery factor (ORF) on displacement fluid flow rate and capillary number is illustrated in Figure 8. It was observed that the oil recovery factor increases with increasing flow rate. Consequently, at a water flow rate of 0.5 µL/min, the recovery factor of the O2 oil sample was 44%, while at a flow rate of 10 µL/min, it reached 70%. As the displacement water flow rate increases, the proportion of residual oil decreases. At flow rates ranging from 0.5 to 10 µL/min, the so-called capillary-pressure displacement mode is achieved. The flow rate of 0.5 µL/min, corresponding to the minimum oil recovery factor, was selected for subsequent experiments.

### 3.2. Effect of Nanoparticle Concentration

**Figure 9:**
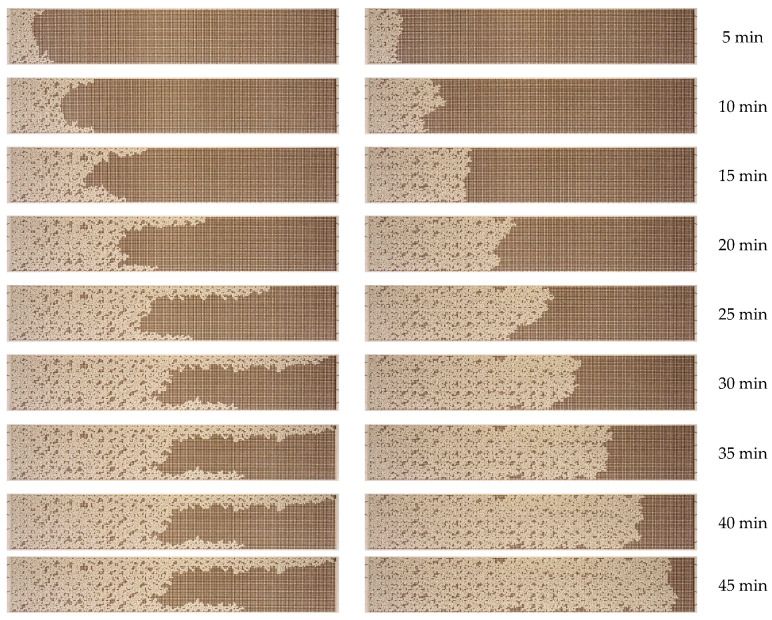

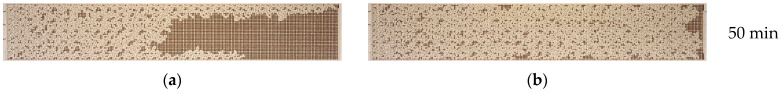
Photographs illustrating oil distribution during flooding: (**a**) with water, and (**b**) with 1030 nanosuspension comprising 10 nm particles at a concentration of 2 wt%.

Subsequently, a series of microfluidic experiments on oil recovery using different nanosuspensions was conducted. The oil sample O2 was used, and a multitude of nanosuspensions were employed as displacement fluids. The flow rate of the nanosuspensions was 0.5 μL/min. The initial step was to investigate the effect of nanoparticle concentration. Figure 9 presents a comparison of oil displacement by water and nanosuspension at various points in time. As illustrated, the displacement process undergoes a transformation when using the nanoparticle suspension. The movement of nanoparticle suspensions occurs in the form of separate jets, similar to the case of using water as a displacement agent; however, the width of these jets increases significantly.

The main difference is that the displacement front in this case becomes significantly more uniform. In the case of displacement by water, the latter quickly rushes to the outlet of the micromodel mainly along the walls. As a result, there is no movement of displacement water in most of the chips, whereas the nanosuspension forms a significantly more uniform moving front. As a result, the breakthrough of the displacement fluid to the outlet from the porous medium of the microfluidic chip occurs significantly later than that when using water. The breakthrough time increases with increasing nanoparticle concentration by approximately 1.5 times. This means that at a given injection flow rate, the volume of the porous medium filled with the displacement nanosuspension increases by about the same number of times. The jets become wider, covering a larger area, which, certainly, leads to a significant enhancement in oil recovery.

**Figure 10:**
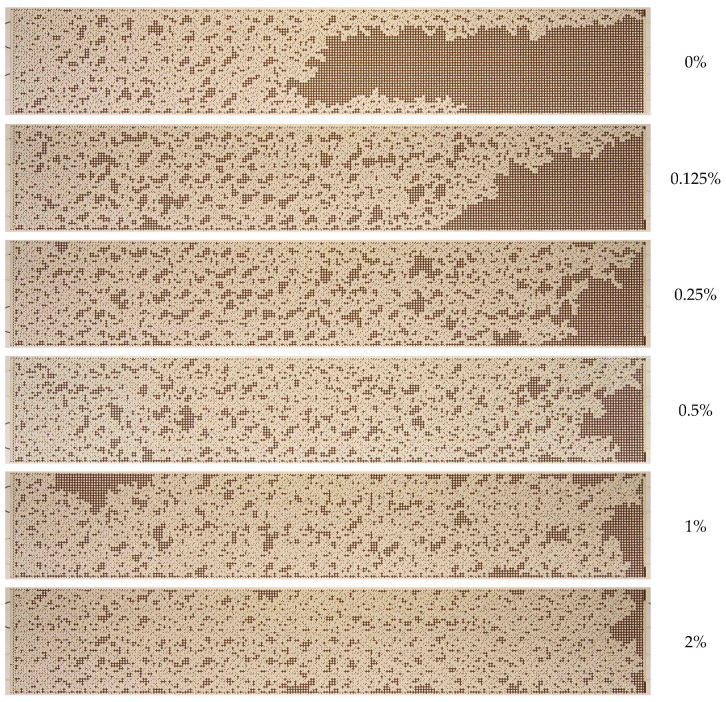
Residual oil saturation after displacement by 1030 nanosuspension with different particle concentrations.

Figure 10 presents the final images of residual oil during displacement with 1030 nanosuspension at varying particle concentrations. As illustrated in Figure 10, the residual oil saturation exhibits a monotonic decrease with increasing nanoparticle concentration.

**Figure 11:**
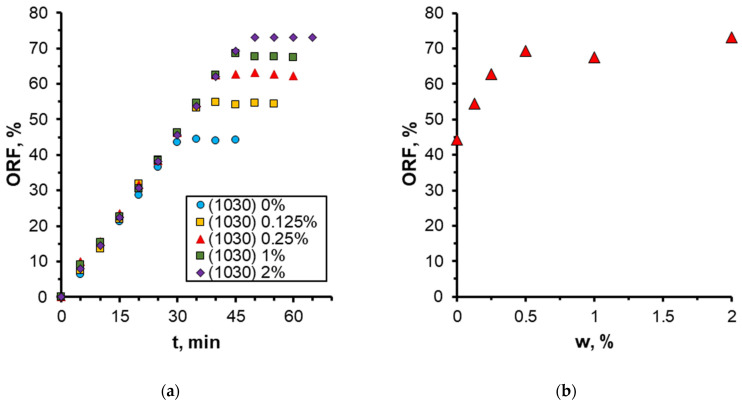
Time behavior of ORF (**a**) and its dependence on nanoparticle weight concentration (**b**) when using 1030 nanosuspension.

The quantitative results on the effect of nanoparticle concentration on the oil recovery factor (ORF) are presented in Figure 11. It was demonstrated that as the nanoparticle concentration increased, the ORF exhibited a corresponding increase, reaching a maximum of 73% at the highest nanoparticle concentration. Conversely, the most pronounced alterations were discernible at particle concentrations below 0.5 wt%. At higher concentrations, no further increment in the oil recovery factor was observed. Therefore, in this case, the concentration of nanoparticles equal to 0.5 wt% should be regarded as the optimal concentration. The analysis of the time behavior of ORF shown in Figure 11a demonstrates that with increasing nanoparticle concentration, not only does its absolute value increase but its time to reach the steady-state value also changes. Thus, while displaced by water, ORF stabilizes approximately 30 min following the commencement of the experiment; at a specific nanoparticle concentration, the stabilization time increases to up to 45 min. As previously stated, this phenomenon can be attributed to the more uniform movement of the displacement front in nanosuspension.

**Figure 12:**
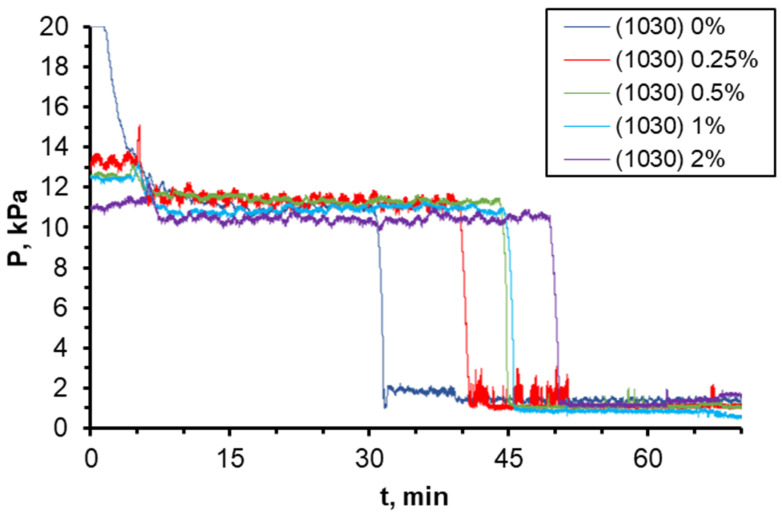
Time dependences of pressure drop in the course of oil displacement by 1030 nanosuspension with different particle concentration.

One of the most significant characteristics of flooding is the injection pressure of displacement fluid. The dependence of pressure at the inlet to the microchip during injection of nanosuspensions with different nanoparticle concentrations is illustrated in Figure 12. A sudden drop in pressure is observed at the moment of breakthrough of the aqueous phase into the chip outlet. As mentioned earlier, due to the more uniform motion of the displacement front when using nanosuspension, the breakthrough time increases with increasing particle concentration. In addition, it is seen that as the concentration of particles increases, the maximum pressure at the beginning of injection decreases due to the fact that the addition of nanoparticles leads to a decrease in the wetting angle and interfacial tension, and as a consequence, a decrease in capillary pressure.

**Figure 13:**
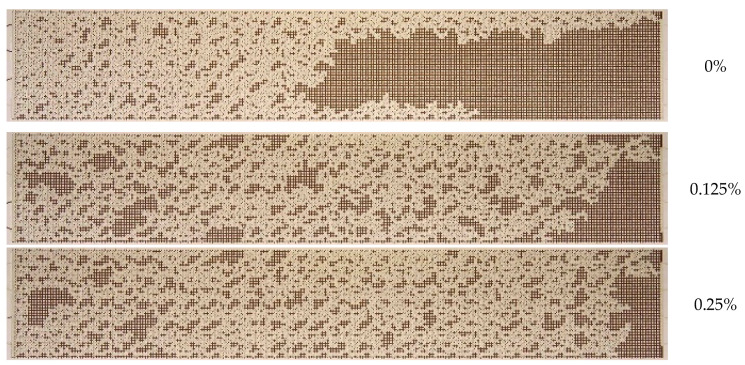

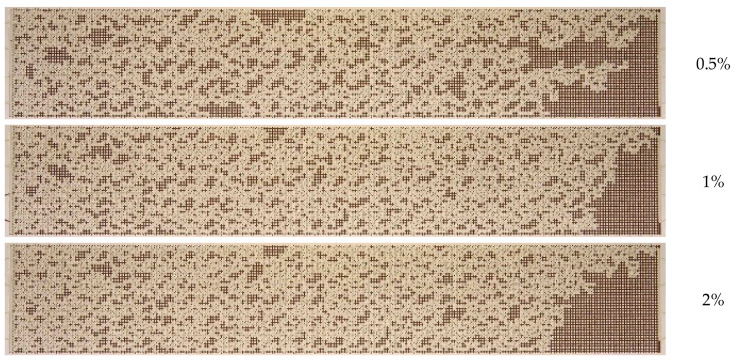
Residual oil saturation after displacement by WA1530 nanosuspension with different particle concentration.

**Figure 14:**
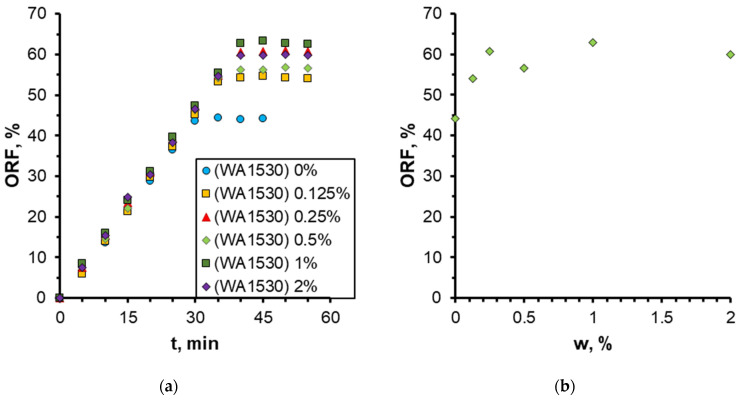
Time behavior of ORF (**a**) and its dependence on nanoparticle concentration (**b**) when using WA1530 nanosuspension.

In general, qualitatively similar results were obtained for WA1530 sol when studying the effect of nanoparticle concentration (Figure 13 and Figure 14). In this case, the silica nanoparticles were treated with a stabilizing aluminum component, and unlike sol 1030 which is alkaline (pH = 9.6), WA1530 sol is acidic (pH = 2.3). The analysis of the photographs of oil fraction distribution and displacement nanosuspensions has demonstrated that the enhancement of oil recovery was observed even at low nanoparticle concentrations of 0.125–0.25%. With increasing concentration, oil recovery generally increased. For acidic sol WA1530, the maximum ORF equal to 65% was observed at a particle concentration of 1 wt%, which is about 8% less than for alkaline sol 1030. It was also revealed that increasing the nanoparticle concentration above 0.5 wt% had almost no effect on further change in ORF. The application of alkaline nanosuspensions to enhance oil displacement has been demonstrated to be more effective than the use of acidic nanosuspensions.

In order to analyze the influencing mechanism of nanoparticle concentration on oil displacement efficiency, one may refer to the results of our recent works [21,22,23], in which the effect of nanoparticle additives on wetting characteristics and interfacial tension was investigated. It has been shown that with increasing concentration of nanoparticles, firstly, the interfacial tension decreases significantly (by 49% at a particle concentration of 2 wt%), and secondly, the wettability with respect to oil changes; the surface becomes hydrophobic. The wetting angle at the oil–water–rock interface increases from 115 to 155° with increasing nanoparticle concentration from 0 to 2 wt%. These processes contribute to the enhanced washing of oil from solid surfaces and the improved washout of capillary-retained oil, which undoubtedly affects the oil recovery factor. Consequently, the oil recovery factor is enhanced when flooding with nanosuspension.

### 3.3. Effect of Nanoparticle Size

**Figure 15:**
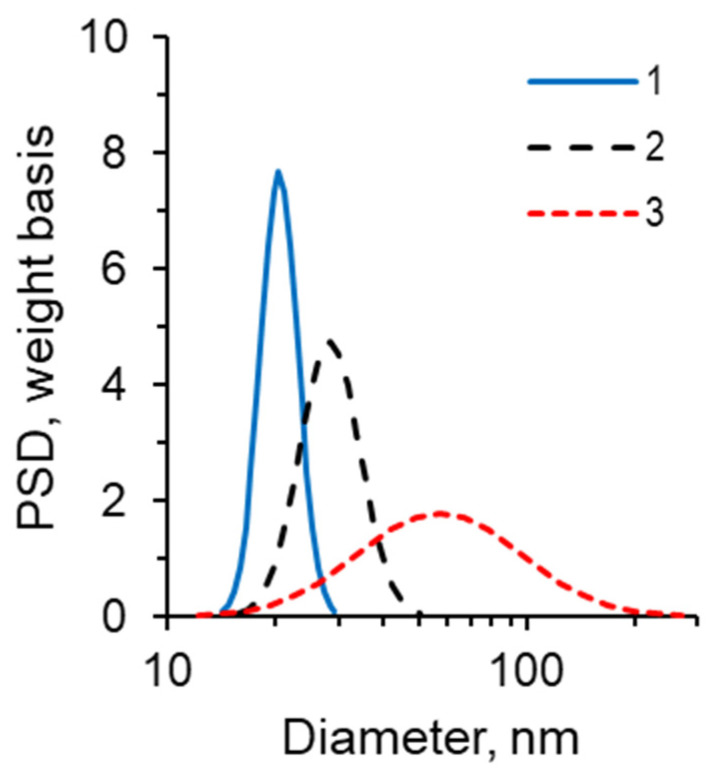
Particle size distributions (PSD) in nanosuspensions: 1—1% 1030 nanosuspension, 2—1% 2040AS nanosuspension, 3—1% 3550 nanosuspension.

Subsequently, a series of microfluidic experiments were conducted to elucidate the impact of nanoparticle size. The O2 oil sample exhibiting the highest viscosity was used for these investigations. Nanosuspensions with markedly disparate average nanoparticle sizes (10, 20, and 35 nm) were employed to displace the oil. The sizes were monitored by electron microscopy (Figure 3) and by measuring particle size distributions directly in the fluid using a DT-1202 acoustic spectrometer (Dispersion Technology Inc., Bedford Hills, NY, USA) (Figure 15).

**Figure 16:**
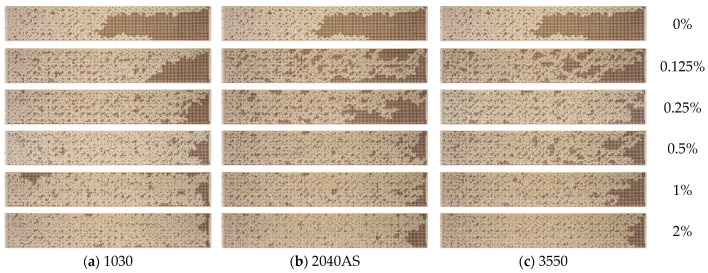
Residual oil saturation after displacement by nanosuspensions with different average particle sizes: (**a**) 10 nm, (**b**) 20 nm, (**c**) 35 nm.

Figure 16 presents a comparison of the residual oil observed in the microfluidic chip when using distinct nanoparticle suspensions at varying concentrations. From a visual analysis of photographs, it is seen that the degree of oil saturation decreases as the average nanoparticle size decreases, provided that all other parameters remain unchanged.

**Figure 17:**
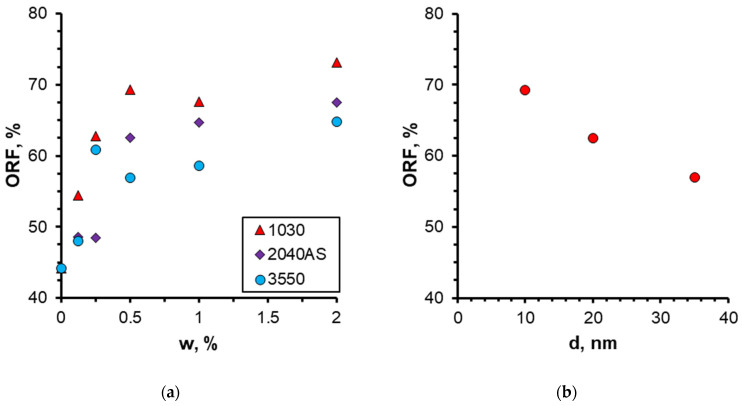
Dependence of oil recovery factor on the concentration of nanoparticles (**a**) and nanoparticle size (**b**) when using 0.5 wt% nanosuspension.

The quantitative impact of nanoparticle size can be observed in Figure 17a, which depicts the oil recovery factor as a function of particle concentration. It can be definitively stated that the utilization of 1030 nanosuspension with a minimum particle size as a displacement fluid results in the most pronounced enhancement of oil displacement (up to 72%). Figure 17b illustrates the experimental dependence of the oil recovery factor on the average nanoparticle size at a fixed concentration of different nanosuspensions. It can be observed that a reduction in nanoparticle size has a beneficial impact on oil displacement. Concurrently, the effect of nanoparticle size is found to be as significant as that of concentration. By reducing the size of nanoparticles from 35 to 10 nm, the ORF increases by almost 15%. This behavior can be attributed to the changes in wettability that nanoparticles of varying sizes exert. As demonstrated in [21], a reduction in nanoparticle size results in a notable enhancement in the nanosuspension hydrophilicity. This enhances the efficacy of the surface flushing from the oil during flooding by nanosuspension.

### 3.4. Effect of Oil Properties

The physical and chemical properties of oil, in particular viscosity, play a significant role in oil recovery efficiency. The oil viscosity coefficient varies considerably from one field to another. However, currently, the question of how nanosuspension will affect the displacement of oil with different viscosity remains insufficiently explored.

To address this question, a series of experiments were conducted to displace different oil samples using 1030 nanosuspension. The flow rate of the nanosuspension was 0.5 µL/min. Three oil samples (O1, O2, and O3) with significantly different viscosity coefficients were investigated. The results are presented in Table 1. The oil samples differed in elemental composition, as well as in density and viscosity coefficient. The sequence of the oil samples, ordered from highest to lowest density and viscosity, is consistent with the following order: O3, O2, and O1. The viscosity of the oil samples ranged from 8.33 to 79.3 mPa × s. 

**Figure 18:**
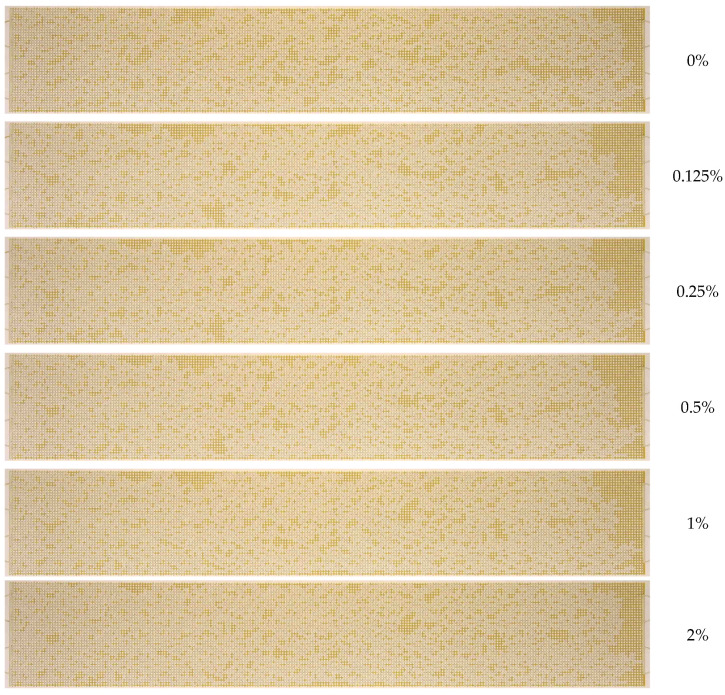
Photographs of the distribution of residual oil (O3) and 1030 nanosuspension at different concentrations.

**Figure 19:**
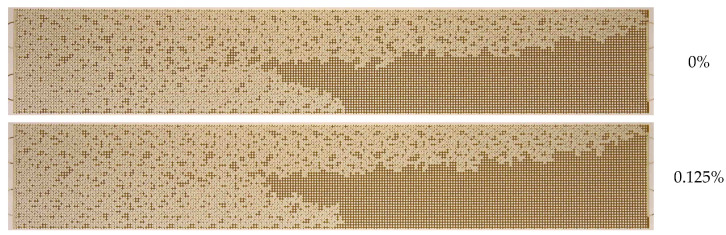

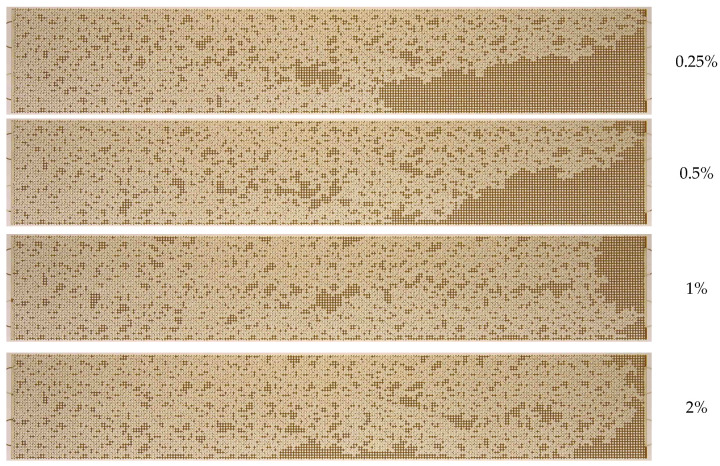
Photographs of the distribution of residual oil (O1) and 1030 nanosuspension at different concentrations.

**Figure 20:**
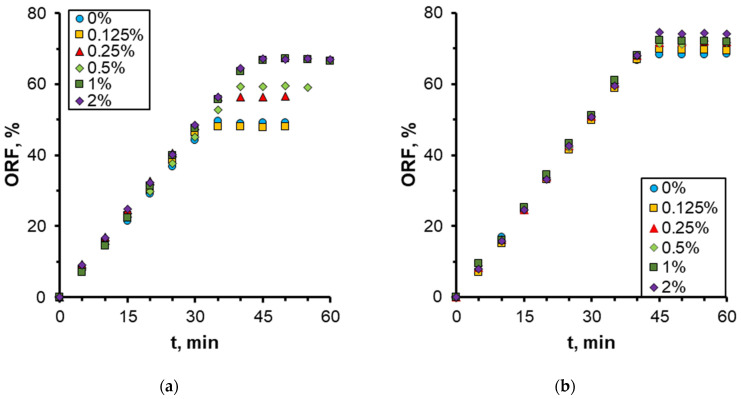
Time behavior of ORF: oil sample O1 (**a**) and O3 (**b**), when using 1030 nanosuspension.

Figure 18, Figure 19 and Figure 20 below illustrate the results of the displacement process of O3 and O1 oil samples, respectively. The analysis of the experimental results shows that ORF significantly decreased as the viscosity of the oil increased. Consequently, for low-viscosity oil, the ORF when using water is approximately 68%, while for high-viscosity oil, it decreases to approximately 45%. It is seen that the lighter O3 oil exhibits a high recovery factor even when displaced by water. The nature of displacement exhibits a notable disparity. The displacement of low-viscosity O3 oil is primarily driven by a uniform front (Figure 18), whereas the displacement of O1 high-viscosity oil by water occurs through the formation of separate streams, resulting in the retention of significant oil-containing regions not affected by the flow.

**Figure 21:**
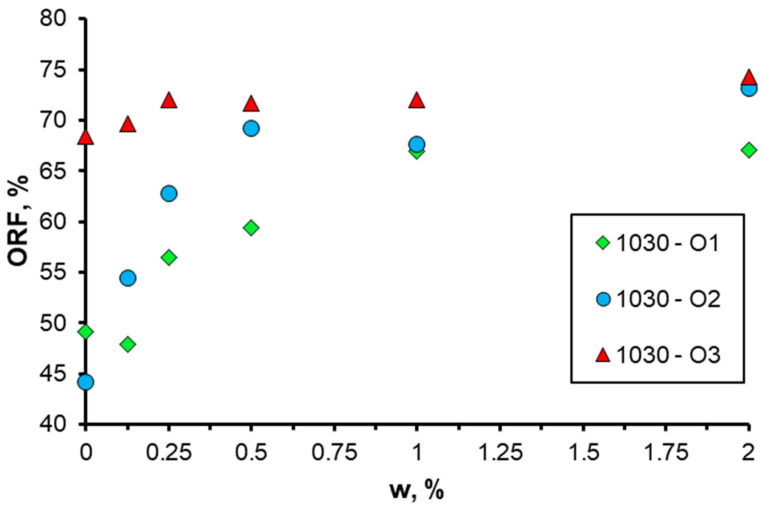
Dependence of recovery factor of different oil samples when using 1030 nanosuspension.

The oil displacement pattern changes significantly with increasing nanoparticle concentration. The results of microfluidic experimental studies have demonstrated that the use of nanosuspensions consistently resulted in an increase in ORF for all oil samples under consideration. Moreover, the ORF increased with an increase in particle concentration. This is illustrated in Figure 21.

However, as is seen, the effect of nanoparticle addition significantly depends also on the oil viscosity. The most significant increment in the ORF is observed for more viscous oils. In this case, the addition of nanoparticles can increase the ORF by more than 20% compared to that of water. With decreasing oil viscosity, its ORF even when displaced by water is high enough so that its further increase from adding nanoparticles is quite insignificant. Thus, for low-viscosity oil, the increment of ORF increasing the concentration of nanoparticles up to 2 wt% was about 6%. Figure 21 shows that the use of nanosuspensions makes oil displacement efficiency weakly dependent on oil viscosity. Figure 21 demonstrates that the use of nanosuspensions does not significantly enhance the oil displacement efficiency, since the latter is only weakly dependent on oil viscosity. Thus, for the light oil O3, when using a 2 wt% nanosuspension, the ORF was 74.3%, while for the heavier and more viscous O2 and O1 oils, the ORF was 73.1 and 67%, respectively. This is a beneficial aspect because it renders the water flooding by nanosuspensions a universal process.

**Figure 22:**
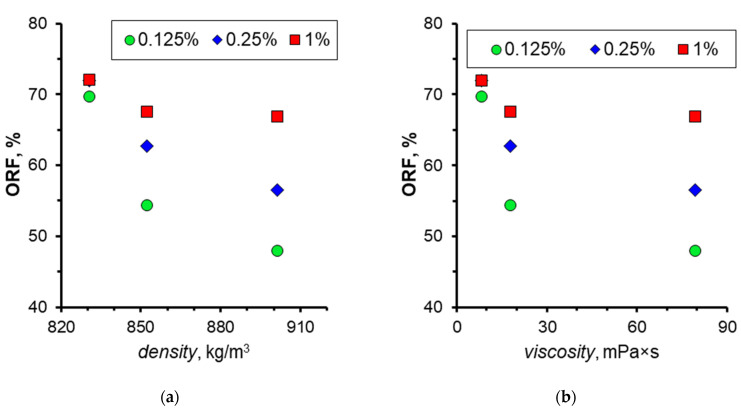
Effect of oil density (**a**) and viscosity coefficient (**b**) on the ORF when using 1030 nanosuspension.

The obtained data permit the construction of a graph representing the ORF at displacement by nanosuspension as a function of the oil density and viscosity. These dependencies are depicted in Figure 22. As illustrated, augmenting the nanoparticle concentration in all cases exerts a beneficial influence on oil displacement. However, this effect is most pronounced in oil samples with high density and high viscosity coefficients. Thus, it was demonstrated that nanosuspensions are most effective in fields with highly viscous oils.

## 4. Conclusions

A systematic study of the effect of nanoparticle addition on the oil displacement efficiency using various nanosuspensions was conducted employing a microfluidic model of a porous medium constructed from quartz glass. A total of approximately a dozen nanosuspensions, prepared by dilution of silica sols in water, were investigated as displacement fluids for the first time. The nanoparticle concentration in the nanosuspensions ranged from 0.125 to 2 wt%, with a corresponding size range of 10 to 35 nm. Moreover, the silica sols under consideration exhibited differences in the composition of functional groups and pH. The effect of concentration, nanoparticle size, fluid flow rate, and the viscosity of the displaced oil has been subjected to investigation.

The following conclusions were drawn.

1. A notable enhancement in oil displacement was observed during flooding with nanosuspensions, even at low nanoparticle concentrations of 0.125–0.25%. The ORF exhibited a notable increase with rising nanoparticle concentration in nanosuspensions. The maximum increase in the ORF compared to water was approximately 28%. Increasing the nanoparticle concentration above 0.5 wt% has practically no further effect on the ORF, and thus, the concentration of 0.5 wt% should be considered optimal.
2. The effect of nanoparticle size on oil recovery from microfluidic chips was investigated. The results demonstrate that the ORF increases as the size of the nanoparticles decreases. The use of 1030-grade nanosuspensions with a minimum nanoparticle size (10 nm) as a displacement fluid results in the greatest enhancement of oil displacement, provided that all other parameters remain constant.
3. The application of alkaline nanosuspensions to enhance oil displacement has been demonstrated to be more effective than the use of acidic nanosuspensions.
4. The impact of oil viscosity on displacement efficiency when using nanosuspensions was examined. The results demonstrate that the presence of nanoparticles in the displacement fluid exerts a favorable influence on the displacement efficiency for all the examined oil samples. Concurrently, the incorporation of nanoparticles to enhance oil displacement exerts the most pronounced impact on high-viscosity oil samples.

Thus, the results of microfluidic experiments have demonstrated that nanosuspensions have significant potential for application in water flooding. Their efficacy is contingent upon the concentration and size of nanoparticles, as well as the composition of functional groups. It is supposed that they will be most effective in formations with more viscous oil.

## Figures and Tables

**Table 1:** Density and viscosity coefficient of the crude oil samples used.

Sample	μ, mPa × s	ρ, kg/m^3^
O1	79.3	901.3
O2	17.8	852.4
O3	8.34	830.6

**Table 2:** Content of elements (mg/kg).

Elements	Samples		
	O1	O2	O3
Ba	19.51	17.58	24.92
Ca	30.86	13.10	122.8
Cl	617.3	62.89	457.1
Mg	-	-	-
Mo	11.86	2.774	11.70
S	2156	6328	2356
Si	12.02	6.174	18.40
Zn	21.28	20.26	21.64

**Table 3:** Optical density and spectral coefficients characterizing the chemical structure of compounds of the studied products.

Sample	Optical Density D at the Maximum of Absorption Bands λ, cm^−1^						Spectral Coefficients				
	1710	1600	1465	1380	1030	720	C1	C2	C3	C4	C5
O1	0.013	0.062	0.867	0.452	0.045	0.096	0.646	0.015	0.521	8.839	0.052
O2	0.011	0.050	0.846	0.445	0.048	0.119	0.420	0.013	0.526	11.280	0.057
O3	0.016	0.033	0.779	0.383	0.035	0.130	0.254	0.021	0.492	15.545	0.045

**Table 4:** Data on the source silica sols for preparing nanosuspensions.

Sample	Manufacturer/Country	Diameter, nm	Concentration, wt%	pH	Stabilization
1030	RusSilica, Russia	10	30	9.6	Na^+^
WA1530	RusSilica, Russia	15	30	2.3	Al component
2040AS	RusSilica, Russia	20	40		
3550	RusSilica, Russia	35	50	8.9	NH_4_^+^

**Table 5:** Physical properties of the used displacement fluids (water W and nanosols).

Name	Concentration, wt%	Viscosity, mPa × s	Density, kg/m^3^
W	0	0.8909	997.0
0.125% 1030	0.125	0.8959	997.7
0.25% 1030	0.25	0.9031	998.4
0.5% 1030	0.5	0.9177	999.7
1% 1030	1	0.9467	1002
2% 1030	2	1.0049	1008
0.125% WA1530	0.125	0.8948	997.7
0.25% WA1530	0.25	0.9014	998.4
0.5% WA1530	0.5	0.9147	999.7
1% WA1530	1	0.9411	1002
2% WA1530	2	0.9940	1008
0.125% 2040AS	0.125	0.8940	997.7
0.25% 2040AS	0.25	0.8994	998.4
0.5% 2040AS	0.5	0.9100	999.7
1% 2040AS	1	0.9313	1002
2% 2040AS	2	0.9740	1008
0.125% 3550	0.125	0.8932	997.7
0.25% 3550	0.25	0.8973	998.4
0.5% 3550	0.5	0.9055	999.7
1% 3550	1	0.9218	1002
2% 3550	2	0.9545	1008

## Data Availability

Data is contained within the article.

## References

[1] Saadat M., Tsai P., Ho T.H., Øye G., Dudek M. (2020). Development of a Microfluidic Method to Study Enhanced Oil Recovery by Low Salinity Water Flooding. ACS Omega.

[2] Lifton V.A. (2016). Microfluidics: An enabling screening technology for enhanced oil recovery (EOR). Lab A Chip.

[3] Gogoi S., Gogoi S.B. (2019). Review on microfluidic studies for EOR application. J. Pet. Explor. Prod. Technol..

[4] Yun W., Chang S., Cogswell D.A., Eichmann S.L., Gizzatov A., Thomas G., Al-Hazza N., Abdel-Fattah A., Wang W. (2020). Toward Reservoir-on-a-Chip: Rapid Performance Evaluation of Enhanced Oil Recovery Surfactants for Carbonate Reservoirs Using a Calcite-Coated Micromodel. Sci. Rep..

[5] Saadat M., Yang J., Dudek M., Tsai P., Øye G., Tsai P.A. (2021). Microfluidic investigation of enhanced oil recovery: The effect of aqueous floods and network wettability. J. Pet. Sci. Technol. Eng..

[6] Pryazhnikov M.I., Minakov A.V., Pryazhnikov A.I., Denisov I.A., Yakimov A.S., Nemtsev I.V. (2021). Application of microfluidic technologies for enhanced oil recovery. J. Phys. Conf. Ser..

[7] Seyyedi M., Sohrabi M. (2020). Oil Reservoir on a Chip: Pore-Scale Study of Multiphase Flow during Near-Miscible CO2 EOR and Storage. Transp. Porous Media.

[8] Tahir M., Liu W., Zhou H., Memon A., Ansari U., Akbar I., Zafar A., Shaikh A., Kashif M., Urinov A. (2020). A review study on micro fluid chips for enhancing the oil recovery by injecting the chemical floods. Therm. Sci..

[9] Fang Z., Cao X.-R., Yu Y.-L., Li M. (2019). Fabrication and Characterization of Microcapsule Encapsulating EOR Surfactants by Microfluidic Technique. Colloids Surf. A.

[10] Hou J., Du J., Sui H., Sun L. (2022). A review on the application of nanofluids in enhanced oil recovery. Front. Chem. Sci. Eng..

[11] Safarzadeh S., Bila A., Torsæter O. (2022). Experimental Investigation of the Effect of Silica Nanoparticles on Interfacial Tension and Wettability during Low Salinity Water Flooding: A Micromodel Study. J. Mod. Nanotechnol..

[12] Tuok L.P., Elkady M., Zkria A., Yoshitake T., Abdelkader S.A., Seyam D.F., El-Moneim A.A., Fath El-Bab A.M.R., Eldemerdash U.N. (2024). Experimental investigation of copper oxide nanofluids for enhanced oil recovery in the presence of cationic surfactant using a microfluidic model. Chem. Eng. J..

[13] Pryazhnikov A.I., Minakov A.V., Pryazhnikov M.I., Zhigarev V.A., Nemtsev I.V. (2022). Displacement of oil by SiO2-nanofluid from a microporous medium: Microfluidic experiments. J. Phys. Conf. Ser..

[14] Rostami P., Sharifi M., Aminshahidy B., Fahimpour J. (2019). The effect of nanoparticles on wettability alteration for enhanced oil recovery: Micromodel experimental studies and CFD simulation. Pet. Sci..

[15] Pryazhnikov M.I., Minakov A.V., Pryazhnikov A.I., Denisov I.A., Yakimov A.S. (2022). Microfluidic Study of the Effect of Nanosuspensions on Enhanced Oil Recovery. Nanomaterials.

[16] (2023). Standard Test Method for API Gravity of Crude Petroleum and Petroleum Products (Hydrometer/Method).

[17] Hennekam R., Sweere T., Tjallingii R., de Lange G.J., Reichart G.-J. (2019). Trace metal analysis of sediment cores using a novel X-ray fluorescence core scanning method. Quat. Int..

[18] Oprea C., Maslov O.D., Gustova M.V., Belov A.G., Szalanski P.J., Oprea I.A. (2009). IGAA and XRF methods used in oil contamination research. Vacuum.

[19] Wanatasanappan V.V., Kanti P.K., Sharma P., Husna N., Abdullah M.Z. (2023). Viscosity and rheological behavior of Al2O3-Fe2O3/water-EG based hybrid nanofluid: A new correlation based on mixture ratio. J. Mol. Liq..

[20] Moshfeghi R., Toghraie D. (2022). An analytical and statistical review of selected researches in the field of estimation of rheological behavior of nanofluids. Powder Technol..

[21] Pryazhnikov M.I., Zhigarev V.A., Minakov A.V., Nemtsev I.V. (2023). Spontaneous imbibition experiments for enhanced oil recovery with silica nanosols. Capillarity.

[22] Davoodi S., Al-Shargabi M., Wood D.A., Rukavishnikov V., Minaev K. (2022). Experimental and field applications of nanotechnology for enhanced oil recovery purposes: A review. Fuel.

[23] Emelyanenko A.M., Boinovich L.B. (2023). The Role of Dispersed Particles in the Physicochemical Behavior of Nanofluids. Colloid J..

